# P/FP ratio: incorporation of PEEP into the PaO_2_/FiO_2_ ratio for prognostication and classification of acute respiratory distress syndrome

**DOI:** 10.1186/s13613-021-00908-3

**Published:** 2021-08-09

**Authors:** Sunitha Palanidurai, Jason Phua, Yiong Huak Chan, Amartya Mukhopadhyay

**Affiliations:** 1 Intensive Care Unit, Alexandra Hospital, National University Health System, 378 Alexandra Road, Singapore, 159964 Singapore; 2 FAST and Chronic Programmes, Alexandra Hospital, National University Health System, Singapore, Singapore; 3 Division of Respiratory & Critical Care Medicine, Department of Medicine, National University Hospital, National University Health System, Singapore, Singapore; 4 Yong Loo Lin School of Medicine, National University of Singapore, Singapore, Singapore; 5 Biostatistics Unit, Yong Loo Lin School of Medicine, National University of Singapore, Singapore, Singapore; 6 Medical Affairs, Alexandra Hospital, Singapore, Singapore

**Keywords:** Acute respiratory distress syndrome, Positive end-expiratory pressure, Mortality, PaO_2_/FiO_2_ ratio

## Abstract

**Background:**

The current Berlin definition of acute respiratory distress syndrome (ARDS) uses the PaO_2_/FiO_2_ (P/F) ratio to classify severity. However, for the same P/F ratio, a patient on a higher positive end-expiratory pressure (PEEP) may have more severe lung injury than one on a lower PEEP.

**Objectives:**

We designed a new formula, the P/FP ratio, incorporating PEEP into the P/F ratio and multiplying with a correction factor of 10 [(PaO_2_*10)/(FiO_2_*PEEP)], to evaluate if it better predicts hospital mortality compared to the P/F ratio post-intubation and to assess the resultant changes in severity classification of ARDS.

**Methods:**

We categorized patients from a dataset of seven ARDS network trials using the thresholds of ≤ 100 (severe), 101–200 (moderate), and 201–300 (mild) for both P/F (mmHg) and P/FP (mmHg/cmH_2_O) ratios and evaluated hospital mortality using areas under the receiver operating characteristic curves (AUC).

**Results:**

Out of 3,442 patients, 1,057 (30.7%) died. The AUC for mortality was higher for the P/FP ratio than the P/F ratio for PEEP levels > 5 cmH_2_O: 0.710 (95% CI 0.691–0.730) versus 0.659 (95% CI 0.637–0.681), *P* < 0.001. Improved AUC was seen with increasing PEEP levels; for PEEP ≥ 18 cmH_2_O: 0.963 (95% CI 0.947–0.978) versus 0.828 (95% CI 0.765–0.891), *P* < 0.001. When the P/FP ratio was used instead of the P/F ratio, 12.5% and 15% of patients with moderate and mild ARDS, respectively, were moved to more severe categories, while 13.9% and 33.6% of patients with severe and moderate ARDS, respectively, were moved to milder categories. The median PEEP and FiO_2_ were 14 cmH_2_O and 0.70 for patients reclassified to severe ARDS, and 5 cmH_2_O and 0.40 for patients reclassified to mild ARDS.

**Conclusions:**

The multifactorial P/FP ratio has a greater predictive validity for hospital mortality in ARDS than the P/F ratio. Changes in severity classification with the P/FP ratio reflect both true illness severity and the applied PEEP strategy.

*Trial registration*: ClinialTrials.gov–NCT03946150.

**Supplementary Information:**

The online version contains supplementary material available at 10.1186/s13613-021-00908-3.

## Introduction

Acute respiratory distress syndrome (ARDS) is a diffuse, inflammatory lung injury caused by multiple etiologies, clinically characterized by severe hypoxemia and bilateral radiographic opacities, and physiologically associated with decreased lung compliance [1]. Hospital mortality remains high, ranging from 30 to 50% depending on the severity of illness [2]. The 2012 Berlin definition by the ARDS Definition Taskforce is widely used to diagnose and categorize the severity of ARDS: a ratio of the partial pressure of arterial oxygen (PaO_2_) to the fraction of inspired oxygen (FiO_2_) (P/F ratio) of ≤ 100, 101–200 and 201–300 mmHg are deemed as severe, moderate, and mild, respectively [3]. Such thresholds have been used to determine management strategies; for example, a higher positive end-expiratory pressure (PEEP) is recommended for moderate and severe, and prone positioning for severe ARDS [1].

The Berlin definition requires a minimum applied PEEP of 5 cmH_2_O, but does not dictate a specific PEEP for the measurement of PaO_2_ [3]. It is, however, well established that PEEP affects PaO_2_ and therefore, P/F ratios with lower PEEP settings result in more patients being labelled as having severe ARDS, and those with higher PEEP settings result in more patients being labelled as having mild or even no ARDS [4–6]. This variability in P/F ratios and classification of severity results in inaccurate prognostication and uncertainty towards when to implement specific therapeutic interventions. A standardized ventilator setting of PEEP ≥ 10 cmH_2_O and FiO_2_ ≥ 0.50, applied at 24 h has thus been suggested to assess severity [7]. Such a wait may, however, lead to a delay in rescue measures and recruitment into clinical trials [8].

In the current study, we hypothesized that since the P/F ratio is intricately tied to PEEP, it may be refined by incorporating PEEP into the formula, thus creating the P/FP ratio while keeping the Berlin definition’s severity classification thresholds of 100, 200, and 300. The aims of our study were to compare the predictive validity for hospital mortality of the P/FP versus the P/F ratio, and to evaluate changes in severity classification from the use of the P/FP rather than the P/F ratio. Some of the results of this study have been published in the form of an abstract in the 2017 American Thoracic Society conference [9].

## Methods

### Data collection

We used the publicly available Biologic Specimen and Data Repository Information Coordinating Centre (BioLINCC) resource and obtained data from a large dataset of seven multicentre randomized controlled trials conducted by the National Heart, Lung, and Blood Institute (NHLBI) ARDS Clinical Trials Network between 1996 and 2013, and published between 1999 and 2014 (Additional file 1: Table E1) [10–16]. These trials enrolled patients with acute lung injury and ARDS as defined by the 1994 American–European Consensus Conference (AECC) definition [17], the predecessor of the Berlin definition. The main difference in the oxygenation criterion between these definitions is the requirement of a minimum PEEP for the latter but not the former. We included patients in all arms of the trials if their initial P/F or P/FP ratios on a PEEP ≥ 5 cmH_2_O post-intubation were ≤ 300 mmHg or 300 mmHg/cmH_2_O, respectively. We excluded patients who were duplicated across trials, as well as patients with missing data for PaO_2_, FiO_2_, and hospital mortality (Additional file 1: Figure E1).

**Table 1:** Patient characteristics, ventilator settings, arterial blood gas results, and outcomes

	All patients (*N *= 3442)
Demographics	
Age, median (IQR), years	51 (39 – 63)
Gender, male (%)	1888 (54.9)
Cause of ARDS,* N* (%)	
Pneumonia	1699 (49.4)
Sepsis	748 (21.7)
Aspiration	429 (12.5)
Trauma	239 (6.9)
Transfusion	71 (2.1)
Others	256 (7.4)

Severity	Severe (≤ 100)			Moderate (101–200)			Mild (201–300)		
Categorization	P/F	P/FP	*P * Value	P/F	P/FP	*P * Value	P/F	P/FP	*P* Value
*N* (%)	640 (19)	794 (30)	< 0.001	1928 (57)	1252 (47)	< 0.001	839 (25)	640 (24)	0.368
Ventilator settings, mean ± SD									
FiO_2_	0.82 ± 0.14	0.79 ± 0.16	< 0.001	0.55 ± 0.14	0.56 ± 0.14	0.049	0.43 ± 0.12	0.49 ± 0.13	< 0.001
PEEP, cmH_2_O	12.6 ± 4.4	14.2 ± 3.6	< 0.001	9.32 ± 3.5	10.2 ± 2.6	< 0.001	8.0 ± 3.6	8.1 ± 2.4	0.544
PaO_2_, mmHg									
Arterial blood gases	65.0 ± 11.4	71.7 ± 19.7	< 0.001	80.0 ± 19.8	81.9 ± 24.9	0.017	104.5 ± 31.2	96.1 ± 38.2	< 0.001
SaO_2_, %	91.2 ± 5.7	92.2 ± 5.4	< 0.001	94.6 ± 3.2	94.5 ± 3.7	0.419	96.9 ± 2.3	95.8 ± 3.0	< 0.001
PaCO_2_, mmHg	43.7 ± 12.9	44.4 ± 13.0	0.309	40.1 ± 9.9	40.3 ± 9.3	0.569	37.8 ± 8.5	38.5 ± 9.0	0.126
pH	7.33 ± 0.10	7.33 ± 0.10	1.000	7.38 ± 0.08	7.38 ± 0.07	1.000	7.39 ± 0.07	7.38 ± 0.07	0.006

P values refer to comparisons between patients classified according to the P/F ratio versus patients classified according to the P/FP ratio in each severity category. Definition of abbreviations: *ARDS*  acute respiratory distress syndrome, *FiO*_*2*_  fraction of inspired oxygen, *IQR*  interquartile range *PaCO*_*2*_  partial pressure of arterial carbon dioxide, *PaO*_*2*_  partial pressure of arterial oxygen, *PEEP* positive end-expiratory pressure, *P/F* =ratio of the PaO_2_ to FiO_2_; P/FP = (PaO_2_ * 10)/(FiO_2_ * PEEP); *SaO*_*2*_  arterial oxygen saturation; *SD* standard deviation

**Fig. 1:**
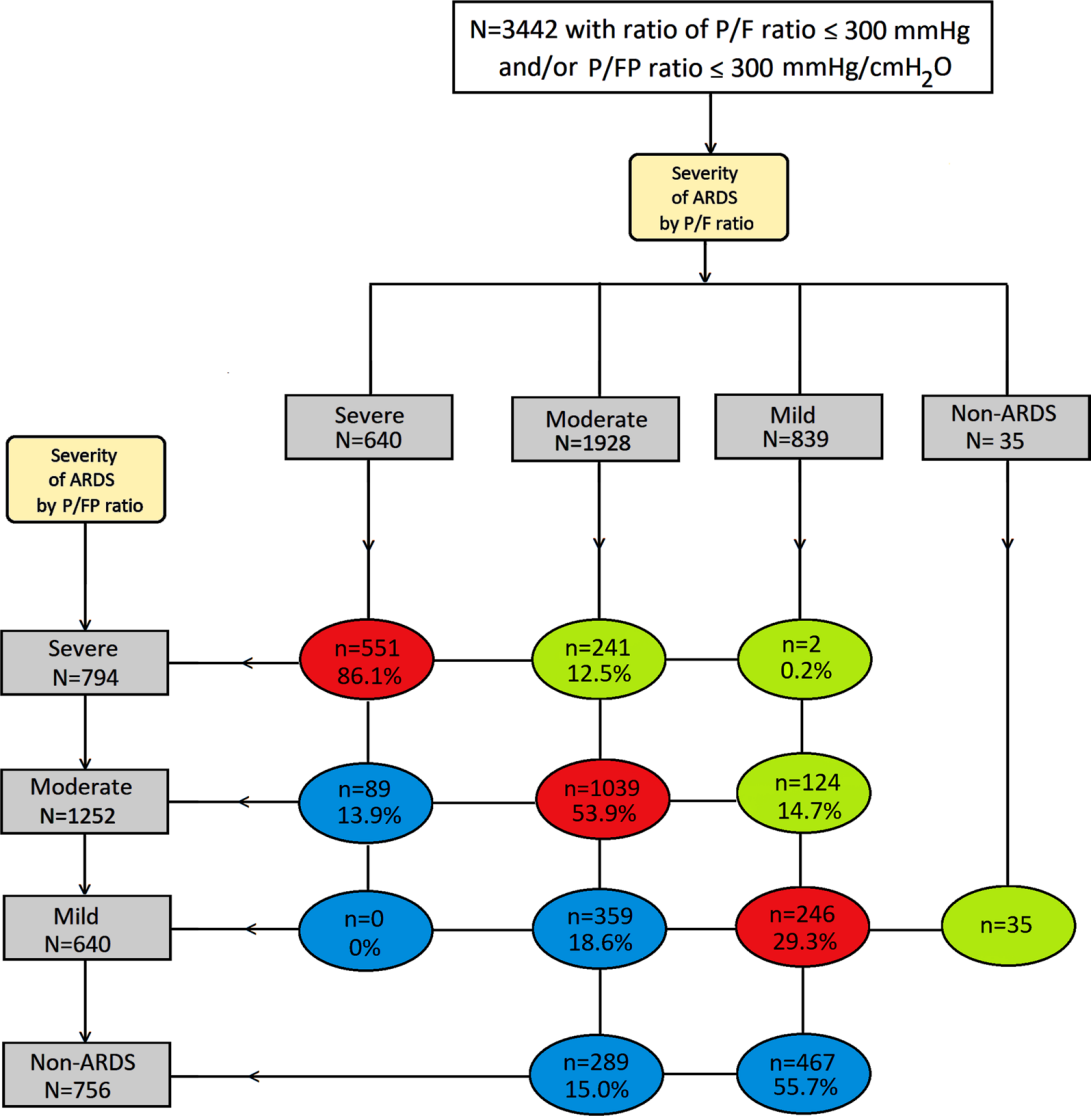
Change of severity classifications when P/FP ratio is used instead of P/F ratio for severities. Severe refers to a ratio of ≤ 100, moderate refers to a ratio of 101–200, mild refers to a ratio of 201–300, non-ARDS refers to a ratio of > 300 mmHg or mmHg/cmH_2_O. Green ovals represent patients who were reclassified to a more severe category. Blue ovals represent patients who were reclassified to a milder category. Red ovals represent patients whose categories remained unchanged. Definition of abbreviations: *ARDS*  acute respiratory distress syndrome; *P/F*  ratio of the partial pressure of arterial oxygen (PaO_2_) to the fraction of inspired oxygen (FiO_2_); *P/FP* = (PaO_2_ * 10)/(FiO_2_ * positive end-expiratory pressure)

We recorded age, gender, causes of ARDS, and the first available arterial blood gas (ABG) measurements (pH, PaO_2_, partial pressure of arterial carbon dioxide [PaCO_2_], and oxygen saturation [SaO_2_]) and corresponding ventilator settings (PEEP and FiO_2_) from day 1 of randomization. The primary outcome measure was hospital mortality.

### P/FP ratio—design and severity classification

All of the seven included trials used a PEEP/FiO_2_ tables for the adjustment of PEEP: five used a low PEEP protocol [10, 12, 14–16], one allowed a low PEEP or a high PEEP protocol or clinician’s discretion [13], and one specifically randomized patients to a low PEEP versus a high PEEP protocol (the ALVEOLI study) [11] (Additional file 1: Table E2). Because of the effect of PEEP on lung recruitment and hypoxemia, we reasoned that while this is certainly not always the case, in general for the same P/F ratio, a patient on a higher PEEP has more severe ARDS than a patient on a lower PEEP. To better reflect the effect of PEEP on P/F ratio, we incorporated it into the denominator, i.e., PaO_2_/(FiO_2_ * PEEP). We then multiplied this by a correction factor of 10, i.e., P/FP ratio = (PaO_2_ * 10)/(FiO_2_ * PEEP), for several reasons. First, there have been previous suggestions to use an applied PEEP of ≥ 10 cmH_2_O as an initial standardized ventilator setting for ARDS [4–7]. Second, a lower average PEEP of 5 to 8 cmH_2_O is more appropriate for non-ARDS surgical and cardiac patients [18, 19]. Third, a regression line plotted for PEEP versus P/F ratio using our dataset intersected the P/F ratio of 150 mmHg (a value midway between 0 and 300 which has been shown to differentiate survivors versus non-survivors reasonably well [20]) at a PEEP of 10 cmH_2_O (Additional file 1: Figure E2.).

**Table 2:** Mortality and ventilator-free days across severity categories

	P/F ratio				P/FP ratio			
Outcomes	Severe (≤ 100) *N = *640	MoNderate (101–200) *N* = 1,928	Mild (201–300)* N* = 839	*P* value for trend	Severe (≤ 100) *N* = 794	Moderate (101–200) *N* = 1,252	Mild (201–300) *N* = 640	*P* value for trend
Hospital mortality, *N* (%)	295 (46.1)	549 (28.5)	204 (24.3)	< 0.001	340 (42.8)	358 (28.6)	161 (25.1)	< 0.001
Ventilator-free days (IQR)	3 (0–17)	17 (0–22)	21 (1.25–24)	< 0.001	1.5 (0–17)	17 (0–22)	19 (0–23)	< 0.001

*P* values refer to comparisons of hospital mortality and ventilator-free days between patients classified as having severe, moderate, and mild ARDS.  *IQR*  interquartile range, *P/F* =ratio of the PaO_2_ to FiO_2_; P/FP = (PaO_2_ * 10)/(FiO_2_ * PEEP)

**Fig. 2:**
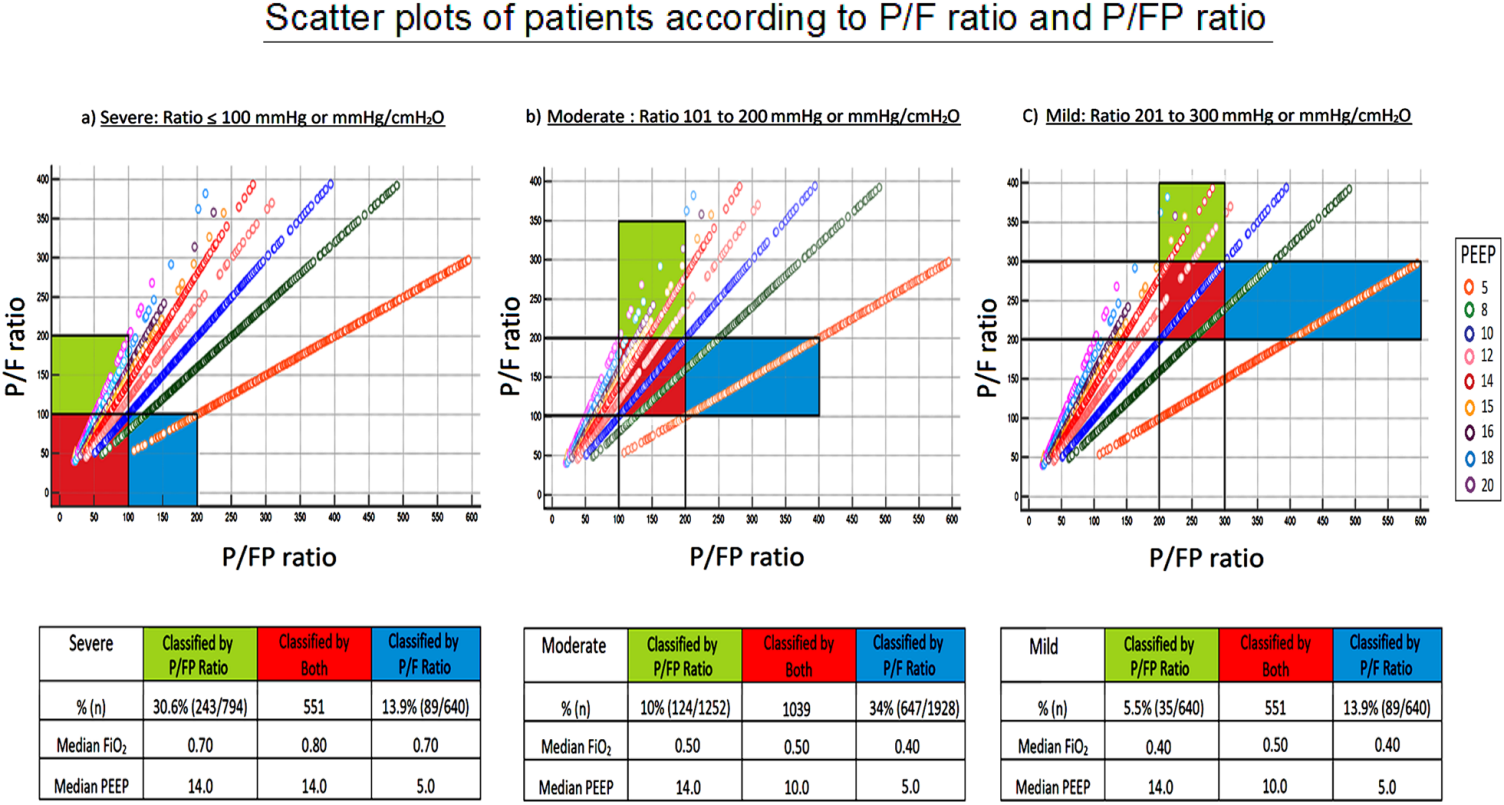
Scatter plots of patients according to P/F ratio and P/FP ratio. Each circle represents individual patients on a certain applied PEEP; each colour represents a different PEEP setting. Green bars represent patients who were classified by the P/FP ratio in each severity. Blue bars represent patients who were classified by the P/F ratio in each severity. Red bars represent patients whose categories remained unchanged. Definition of abbreviations: FiO_2_ = fraction of inspired oxygen; PEEP = positive end-expiratory pressure; P/F = ratio of the partial pressure of arterial oxygen (PaO_2_) to FiO_2_; P/FP = (PaO_2_ * 10)/(FiO_2_ * PEEP)

We used the Berlin definition’s thresholds of ≤ 100, 101–200, and 201–300 to differentiate severe, moderate and mild ARDS, respectively, for both the P/F (mmHg) and the P/FP (mmHg/cmH_2_O) ratios. For greater discrimination, and in view of previous studies which used 150 mmHg as a threshold for prognostication and rescue measures [20–23], we further divided the moderate category into moderate^severe^ (101–150 mmHg or mmHg/cmH_2_O) and moderate^mild^ (151–200 mmHg or mmHg/cmH_2_O) as a secondary analysis. We classified patients who had either a P/F ratio > 300 mmHg or a P/FP ratio ≥ 300 mmHg/cmH_2_O in a non-ARDS category.

### Statistical analyses

We expressed categorical variables as number (%) and continuous variables as mean (standard deviation [SD]) or median (interquartile ranges [IQR]) depending on the observed distribution. We made comparisons using the Chi-square test, analysis of one-way variance, and Kruskal–Wallis test, as appropriate. For the classification of severity of oxygenation, we drew scatter plots with the P/F ratio on the y-axis and the P/FP ratio on the x-axis to reflect the overlap or lack thereof of patients in the various severity classifications as defined by the two ratios. We generated three plots on the severe, moderate, and mild categories as the primary analysis and four plots to include the severe, moderate^severe^, moderate^mild^, and mild categories as a secondary analysis. We overlay circles on these plots to represent individual patients with selected PEEP levels. We then calculated the median PEEP and FiO_2_ for the respective severities.

We constructed receiver operating characteristic (ROC) curves and measured the areas under the curves (AUC) with 95% confidence intervals (CI) to evaluate the predictive validity of the P/F and P/FP ratios for hospital mortality. We treated P/F and P/FP ratios as continuous independent variables and mortality as a binary variable. We performed multiple stratified analyses of mortality at different PEEP levels (≥ 5, > 5, > 8, > 10, > 12, > 14, > 16, > 18 cmH_2_O), thus generating individual AUC for each stepwise increase and compared the various AUC using the Stata *roccomp* command. We calculated the optimal sensitivity and specificity for various thresholds of P/F and P/FP ratios using the Youden index. All analyses were 2-sided, with a *P* value of < 0.05 considered statistically significant, except for the AUC at different PEEP settings, for which corrections for the multiple comparisons were made by setting the significance threshold at < 0.01. We conducted the analyses using MedCalc Statistical Software Version 19.4 (MedCalc Software Ltd., Ostend, Belgium), SPSS Statistics for Windows Version 20.0 (IBM Corp., Armonk, New York), and Stata Statistical Software Release 16 (StataCorp LLC., College Station, Texas). The Domain Specific Review Board, National Healthcare Group, Singapore provided ethics approval for the study (reference number 2017/00325).

## Results

A total of 3,442 patients with either P/F or P/FP ratio less than or equal to 300 were included in the study: 3,407 patients had a P/F ratio of ≤ 300 mmHg, while 2,686 patients had a P/FP ratio of ≤ 300 mmHg/cmH_2_O. Table 1 summarizes their baseline characteristics, ventilator settings, ABG results, and outcomes. The majority (54.9%) were male with a median age of 51 (39–63) years and pneumonia (49.4%) was the leading cause of ARDS. The hospital mortality rate was 30.7% (1,057 deaths). Table 2 shows the progressive increase in mortality rates and decrease in ventilator-free days with worsening severity categories according to both the P/F and P/FP ratios.

### Classification of severity by the P/FP ratio

Figure 1 describes the change in severity classifications when the P/FP ratio was used instead of the P/F ratio. In total 1,860 (54.0%) patients were either moved to a more severe category (green ovals) or to a milder category (blue ovals). As a result, the number of patients in the severe and non-ARDS groups increased, while those in the moderate and mild groups decreased. Specifically, the proportions of patients reclassified to a more severe category (green ovals) were 12.5% of the moderate group and 15.0% of the mild group. An additional 35 patients in the non-ARDS group were moved to the mild category. The proportions of patients reclassified to a milder category (blue ovals) were 13.9% of the severe group and 33.6% of the moderate group. An additional 467 patients in the mild group were moved to the non-ARDS group.

Figure 2 scatterplot shows the percentage of patients in each severity category with their respective median PEEP and FiO_2_. As reflected by the median PEEP levels and FiO_2_, patients who were placed in the same severity categories (red bars) by both ratios were generally given a PEEP consistent with that of the low PEEP protocol. Patients who were reclassified to a more severe category by the P/FP ratio (green bars) were generally given a higher PEEP and FiO_2_, but still being consistent with the low PEEP protocol. On the other hand, patients who were reclassified to a milder category by the P/FP ratio (blue bars) were sometimes given a lower PEEP than that of the low PEEP protocol. Specifically, in the group classified as severe using the P/F ratio and moderate using the P/FP ratio, the median PEEP was only 5 cmH_2_O despite a high median FiO_2_ of 0.70 (the corresponding applied PEEP on the PEEP/FiO_2_ table of a low PEEP strategy should be 10–14 cmH_2_O). In the group classified as mild using the P/F ratio and non-ARDS using the P/FP ratio, ventilator settings were minimal, with a median PEEP of 5 cmH_2_O and a median FiO_2_ of 0.40.

Additional file 1: Figures E3 and E4 also describe the change in severity classifications when the P/FP ratio was used instead of the P/F ratio and the respective median PEEP and FiO_2_, but with greater discrimination by dividing the moderate category into moderate^severe^ (101–150) and moderate^mild^ (151–200): 11.8% of patients from the moderate^mild^ group were reclassified as moderate^severe^, while 14.4% of patients from the moderate^severe^ group were reclassified as moderate^mild^. Additional file 1: Figures E5 and E6, respectively, show the changes in severity classifications in the low and high PEEP arms of the ALVEOLI study [11]. While trends in the low PEEP arm were broadly similar to those of the combined dataset of all studies, more patients in the high PEEP arm were reclassified to the severe category than to the mild category.

### Predictive validity for mortality

**Fig. 3:**
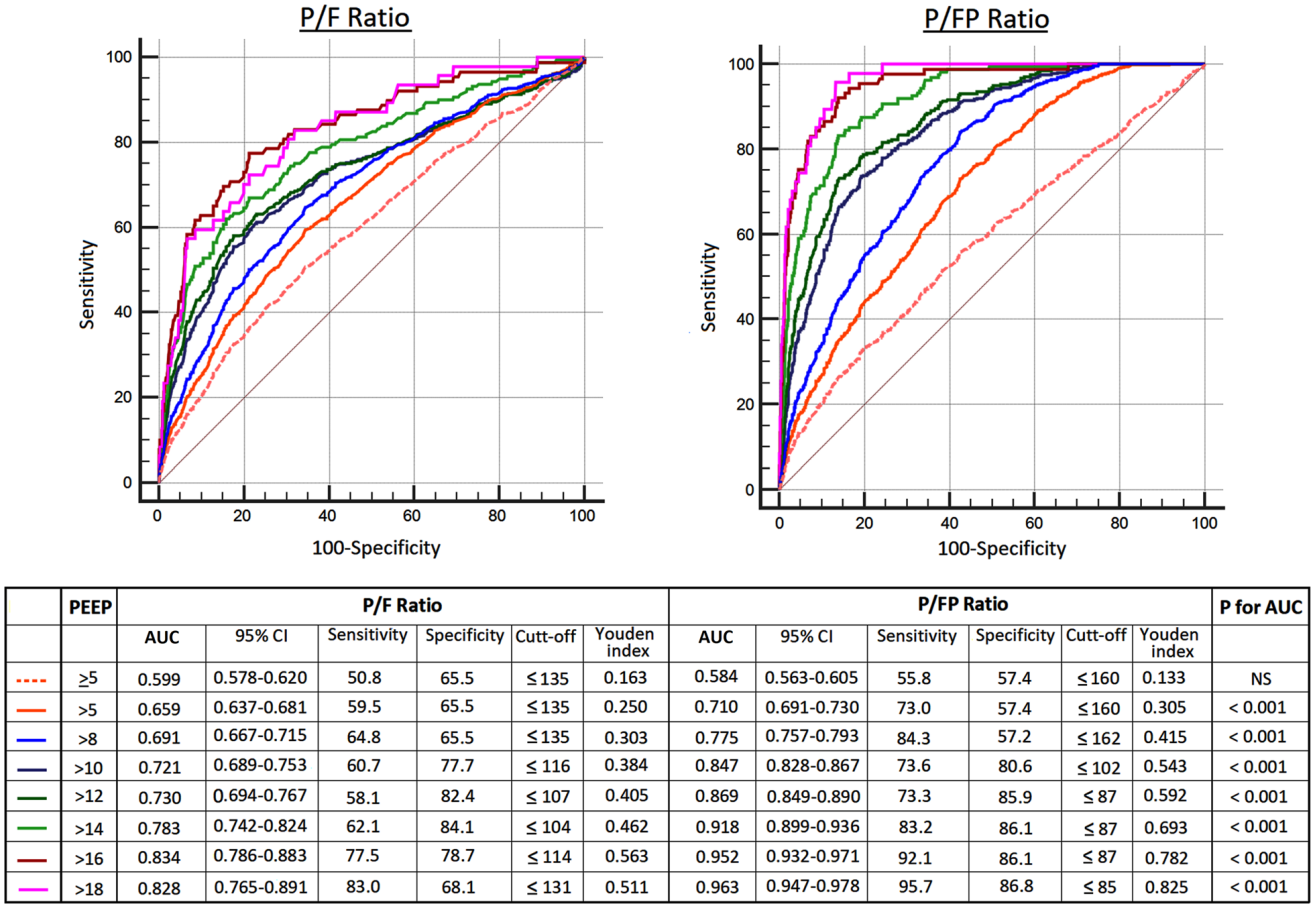
Receiver operating characteristic curves for the P/F ratio and the P/FP ratio for hospital mortality at different PEEP thresholds. *AUC* area under the curve; *NS* not significant, *PEEP* positive end-expiratory pressure, *P/F* = ratio of the partial pressure of arterial oxygen (PaO_2_) to the fraction of inspired oxygen (FiO_2_); P/FP = (PaO_2_ * 10)/(FiO_2_ * PEEP); *CI* confidence interval

Figure 3 shows the ROC curves for the predictive validity of the P/F ratio and the P/FP ratio for hospital mortality. The AUC were significantly higher for the P/FP ratio compared to the P/F ratio with all thresholds of increasing PEEP levels starting from > 5 cmH_2_O (*P* < 0.001). Beyond a PEEP > 5 cmH_2_O, the predictive validity of the P/FP ratio increased with higher PEEP, as evidenced by a significant rise in the AUC between thresholds; this was not seen with the P/F ratio (Additional file 1: Table E3). Correspondingly, as PEEP levels increased, there were greater increases in the sensitivity and specificity of optimal cut-offs for the P/FP ratio based on the Youden index than those for the P/F ratio (Fig. 3). For PEEP > 5 cmH_2_O, the respective AUC for the P/FP ratio and P/F ratio were 0.710 (95% CI 0.691–0.730, sensitivity 73.0% and specificity 57.4% with a P/FP cut-off of 160 mmHg/cmH_2_O) versus 0.659 (95% CI 0.637–0.681, sensitivity 59.5% and specificity 65.5% with a P/F cut-off of 135 mmHg) (*P* < 0.001). For PEEP > 18 cmH_2_O, the respective AUC for the P/FP ratio and P/F ratio were 0.963 (95% CI 0.947–0.978, sensitivity 95.7% and specificity 86.8% with a P/FP cut-off of 85 mmHg/cmH_2_O) versus 0.828 (95% CI 0.765–0.891, sensitivity 83.0% and specificity 68.1% with a P/F cut-off of 131 mmHg) (*P* < 0.001).

## Discussion

This analysis of more than 3,000 patients found that incorporation of PEEP into the P/F ratio increases the predictive validity for hospital mortality in ARDS for those treated with a PEEP of > 5 cmH_2_O. The predictive validity of P/FP ratio improved with progressively higher levels of PEEP. More than half of the patients were reclassified into a different severity category by the P/FP ratio, compared to the P/F ratio. Patients who were reclassified to a more severe category had a relatively high PEEP and FiO_2_, albeit with a mix of settings consistent with both the high and low PEEP protocols. Those who were reclassified to a milder category were given lower ventilator settings, often with minimal PEEP and FiO_2_, sometimes with inadequate PEEP levels lower than that of the low PEEP protocol.

Mortality increases with worsening severity as categorized by the Berlin definition [2, 3]. However, mixed results from various comparative studies make it unclear whether the Berlin criteria outperform the older AECC classification for prognostication [3, 24–26]. This is unsurprising given that other than the former requiring a minimum PEEP of 5 cmH_2_O, the oxygenation criteria for both definitions are essentially similar [3, 17]. Indices such as the P/F ratio and by extension the P/FP ratio were not originally designed to predict mortality. Nonetheless, we found that the AUC of the P/FP ratio for hospital mortality was significantly greater than that of the P/F ratio for all thresholds of PEEP > 5 cmH_2_O. While it must be acknowledged that the AUC of 0.710 for a PEEP > 5 cmH_2_O is not remarkably high, this should be interpreted in context. First, the P/FP ratio was measured on the day of intubation when prognostication is more difficult compared to on subsequent days [24]. Second, the AUC progressively increased with each higher threshold of PEEP, with both sensitivity and specificity of the optimal P/FP cut-offs exceeding 80% with a PEEP > 14 cmH_2_O.

Adding PEEP to the P/F ratio allows consideration of respiratory system compliance and lung recruitment when assessing, firstly the presence and secondly the severity of ARDS. Jardin and colleagues had already attempted to use a combination of PEEP, FiO_2_, and PaO_2_ to predict the progression of ARDS in 1982, but their study included only 50 patients [27]. More recently, an autopsy study found that more than half of patients deemed to have ARDS by the Berlin definition did not actually have diffuse alveolar damage, especially for those meeting the clinical criteria for less than 72 h [28]. This finding was consistent with a clinical study by Villar and colleagues, which showed that on standardized ventilator settings and recruitment with a PEEP of ≥ 10 cmH_2_O, a large proportion of patients were moved to a milder severity category after 24 h, with some no longer fulfilling criteria for ARDS [4–6]. Our study found superior prognostication for the P/FP ratio compared to the P/F ratio despite not waiting 24 h. Beyond PEEP, while others have added various indices of pressure to the P/F ratio to better predict outcomes, such as a score incorporating plateau pressure and the oxygenation index (FiO_2_/[PaO_2_*mean airway pressure]), airway pressures are not easily measured in non-paralyzed patients (24, 29). Notably, we used a multiplication factor of 10 to derive the P/FP ratio for reasons already stated in the Methods section. As illustrated in Additional file 1: Figure E7, multiplication by a lower factor such as 8 will result in more patients classified in the severe category while multiplication by a higher factor such as 12 will result in more patients classified in the mild category. The predictive validity of the ratio as determined by the AUC, however, will remain unchanged regardless of the correction factor.

Although the concept of personalized PEEP settings continues to generate much interest [30], the seemingly generic PEEP/FiO_2_ table (using a high PEEP protocol) does provide appropriately higher PEEP for patients with more severe ARDS and higher recruitability, and lower PEEP for those with less severe ARDS and lower recruitability [31]. Based on evidence suggesting possible survival benefits, clinical practice guidelines from the American Thoracic Society, the European Society of Intensive Care Medicine, and the Society of Critical Care Medicine in 2017 provide a conditional recommendation for higher PEEP settings for moderate and severe ARDS [1]. This notwithstanding, PEEP settings used for severe ARDS in routine practice worldwide centre around a relatively low value of 8.5 cmH_2_O [2]. Although the patients included in our study were enrolled in RCTs which mostly used the low PEEP protocol, some were randomized to the high PEEP protocol in the ALVEOLI study. Given the effects of PEEP on oxygenation, the P/F ratio for any given patient and thus the perceived severity of ARDS will change depending on the applied PEEP strategy.

In this context, the clinical utility of the P/FP ratio may be postulated. First, given its higher predictive validity for mortality compared to the P/F ratio, patients with a low P/FP ratio are more likely to have truly severe ARDS in which oxygenation remains poor despite high PEEP settings. One should, however, evaluate if the applied PEEP is inappropriately high, over and beyond that recommended by the PEEP protocols, in which case the P/FP ratio will be made spuriously low. Second, patients with a high P/FP ratio are likely to have mild ARDS or even no ARDS. However, how the P/FP ratio will change with adjustment of several other facets of ARDS management, particularly titration of PEEP and prone positioning to recruit collapsed lung units and minimize driving pressure, remains unknown. More research will be necessary to investigate these areas of interest and better understand the clinical usefulness of the P/FP ratio. Crucially, current recommendations for various therapeutic interventions for ARDS are mostly derived from trials using the P/F ratio [1]. Further studies are needed before the initiation of these treatments may be tied to P/FP thresholds.

To the best of our knowledge, this is the first validation of a novel index of severity for ARDS that uses easily available parameters and intuitive classification thresholds that are similar to the Berlin definition [3]. We used a large dataset with accurate ABG values and ventilator settings to ensure relevance across a wide range of severity; the results should therefore be relevant to most patients with ARDS. Our study, however, has several limitations. First, the dataset had more patients on a low PEEP than a high PEEP protocol. To better understand how the PEEP strategy affects the P/FP ratio, we performed subgroup analyses of patients from the two arms of the ALVEOLI study [11]. Second, all patients were from RCTs with heterogeneous aims and which may not reflect real-world practice. Regardless, the full range of PEEP settings allowed the calculation of clinically useful P/FP ratios. Third, since the P/FP ratio was internally derived through computing from the P/F ratio and applied PEEP, its ability to predict mortality may decrease in external validation cohorts. On the other hand, we only assessed the P/FP ratio on the day of intubation, and it is possible, though unproven, that its prognostic ability will improve over the next 24 to 72 h. Fourth, while we applied a correction factor of 10 for the PEEP used within the P/FP ratio for reasons already stated, there ultimately remains some degree of arbitrariness as applied PEEP levels vary according to many factors other than oxygenation.

In conclusion, the multifactorial P/FP ratio has a greater predictive validity for mortality in ARDS for patients on a PEEP of > 5 cmH_2_O than the P/F ratio. Its prognostic ability progressively increases with higher levels of PEEP. Changes in severity classification when the P/FP ratio is used instead of the P/F ratio reflect both true illness severity and the applied PEEP strategy.

## Supplementary Information

**Additional file 1: Table E1.** Number of patients included from seven National Heart, Lung, and Blood Institute (NHLBI) ARDS Clinical Trials Network studies.** Table E2.** Low and high PEEP protocols used in included studies.** Table E3.** Pairwise comparisons of areas under the receiver operating characteristic curves for different PEEP thresholds.** Figure E1.** Included patients from seven National Heart, Lung, and Blood Institute (NHLBI) ARDS Clinical Trials Network studies.** Figure E2.** Regression line of PEEP versus P/F ratio.** Figure E3.** Change of severity classifications when P/FP ratio is used instead of P/F ratio.** Figure E4.** Scatter plots for proportion of patients according to P/F ratio and P/FP ratio.** Figure E5.** Change of severity classifications when P/FP ratio is used instead of P/F ratio in the low PEEP arm of the ALVEOLI study.** Figure E6.** Change of severity classifications when P/FP ratio is used instead of P/F ratio 10 in the high PEEP arm of the ALVEOLI study.** Figure E7.** Comparison of P/FP ratios using different correction factors.

## Data Availability

Large clinical dataset of seven multicentre randomized controlled trials conducted by the National Heart, Lung, and Blood Institute (NHLBI) ARDS Clinical Trials Network.
