# Supplementary material for: P/FP ratio: incorporation of PEEP into the PaO2/FiO2 ratio for prognostication and classification of acute respiratory distress syndrome
Source: Ann Intensive Care. 2021 Aug 9;11:124. doi: 10.1186/s13613-021-00908-3 (PMC8350287; doi:10.1186/s13613-021-00908-3)
Supplement: Supplementary file 1 — Additional file 1: Table E1. Number of patients included from seven National Heart, Lung, and Blood Institute (NHLBI) ARDS Clinical Trials Network studies. Table E2. Low and high PEEP protocols used in included studies. Table E3. Pairwise comparisons of areas under the receiver operating characteristic curves for different PEEP thresholds. Figure E1. Included patients from seven National Heart, Lung, and Blood Institute (NHLBI) ARDS Clinical Trials Network studies. Figure E2. Regression line of PEEP versus P/F ratio. Figure E3. Change of severity classifications when P/FP ratio is used instead of P/F ratio. Figure E4. Scatter plots for proportion of patients according to P/F ratio and P/FP ratio. Figure E5. Change of severity classifications when P/FP ratio is used instead of P/F ratio in the low PEEP arm of the ALVEOLI study. Figure E6. Change of severity classifications when P/FP ratio is used instead of P/F ratio 10 in the high PEEP arm of the ALVEOLI study. Figure E7. Comparison of P/FP ratios using different correction factors. [file 13613_2021_908_MOESM1_ESM.docx]

**P/FP ratio - Incorporation of PEEP into the PaO_2_/FiO_2_ ratio for**

**prognostication and classification of acute respiratory distress syndrome**

**Supplementary Appendix**

Sunitha Palanidurai, Jason Phua, Yiong Huak Chan, Amartya Mukhopadhyay

**Contents Page**

**Table E1**. Number of patients included from seven National Heart, Lung, and Blood Institute 2

(NHLBI) ARDS Clinical Trials Network studies

**Table E2**. Low and high PEEP protocols used in included studies     3

**Table E3**. Pairwise comparisons of areas under the receiver operating characteristic curves 4

for different PEEP thresholds

**Figure E1**. Included patients from seven National Heart, Lung, and Blood Institute (NHLBI) 5

ARDS Clinical Trials Network studies

**Figure E2**. Regression line of PEEP versus P/F ratio 6

**Figure E3**. Change of severity classifications when P/FP ratio is used instead of P/F ratio 7

**Figure E4**. Scatter plots for proportion of patients according to P/F ratio and P/FP ratio 8

**Figure E5**. Change of severity classifications when P/FP ratio is used instead of P/F ratio 9 in the low PEEP arm of the ALVEOLI study

**Figure E6**. Change of severity classifications when P/FP ratio is used instead of P/F ratio 10 in the high PEEP arm of the ALVEOLI study

**Figure E7**. Comparison of P/FP ratios using different correction factors 11

**Table E1. Number of patients included from seven National Heart, Lung, and Blood Institute (NHLBI) ARDS Clinical Trials Network studies**

| **Study** | **N** |
| --- | --- |
| ARMA [1] | 792 |
| ALVEOLI [2] | 466 |
| FACTT [3] | 823 |
| ALTA [4] | 227 |
| Omega [5] | 170 |
| EDEN [6] | 461 |
| SAILS [7] | 503 |
| Total | 3,442 |

**References**

1. Acute Respiratory Distress Syndrome Network, Brower RG, Matthay MA, Morris A, Schoenfeld D, Thompson BT, Wheeler A. Ventilation with lower tidal volumes as compared with traditional tidal volumes for acute lung injury and the acute respiratory distress syndrome. *N Engl J Med* 2000;342:1301-1308.
2. Brower RG, Lanken PN, MacIntyre N, Matthay MA, Morris A, Ancukiewicz M, Schoenfeld D, Thompson BT, National Heart, Lung, and Blood Institute ARDS Clinical Trials Network. Higher versus lower positive end-expiratory pressures in patients with the acute respiratory distress syndrome. *N Engl J Med* 2004;351:327-336.
3. National Heart, Lung, and Blood Institute Acute Respiratory Distress Syndrome Clinical Trials Network, Wiedemann HP, Wheeler AP, Bernard GR, Thompson BT, Hayden D, deBoisblanc B, Connors AF Jr, Hite RD, Harabin AL. Comparison of two fluid-management strategies in acute lung injury. *N Engl J Med* 2006;354:2564-2575.
4. National Heart, Lung, and Blood Institute Acute Respiratory Distress Syndrome Clinical Trials Network, Matthay MA, Brower RG, Carson S, Douglas IS, Eisner M, Hite D, Holets S, Kallet RH, Liu KD, MacIntyre N, Moss M, Schoenfeld D, Steingrub J, Thompson BT. Randomized, placebo-controlled clinical trial of an aerosolized beta(2)-agonist for treatment of acute lung injury. *Am J Respir Crit Care Med* 2011;184:561-568.
5. Rice TW, Wheeler AP, Thompson BT, deBoisblanc BP, Steingrub J, Rock P, NIH NHLBI Acute Respiratory Distress Syndrome Network of Investigators. Enteral omega-3 fatty acid, gamma-linolenic acid, and antioxidant supplementation in acute lung injury. *JAMA* 2011;306:1574-1581.
6. National Heart, Lung, and Blood Institute Acute Respiratory Distress Syndrome Clinical Trials Network, Rice TW, Wheeler AP, Thompson BT, Steingrub J, Hite RD, Moss M, Morris A, Dong N, Rock P. Initial trophic vs full enteral feeding in patients with acute lung injury: the EDEN randomized trial. *JAMA* 2012;307:795-803.
7. National Heart, Lung, and Blood Institute ARDS Clinical Trials Network, Truwit JD, Bernard GR, Steingrub J, Matthay MA, Liu KD, Albertson TE, Brower RG, Shanholtz C, Rock P, Douglas IS, deBoisblanc BP, Hough CL, Hite RD, Thompson BT. Rosuvastatin for sepsis-associated acute respiratory distress syndrome. *N Engl J Med* 2014;370:2191-2200.

**Table E2. Low and high PEEP protocols used in included studies**

| Low PEEP protocol used in the ARMA, FACTT, Omega, EDEN, SAILS, and ALVEOLI study | | | | | | | | | | | | | | |
| --- | --- | --- | --- | --- | --- | --- | --- | --- | --- | --- | --- | --- | --- | --- |
| FiO_2_ | 0.3 | 0.4 | 0.4 | 0.5 | 0.5 | 0.6 | 0.7 | 0.7 | 0.7 | 0.8 | 0.9 | 0.9 | 0.9 | 1.0 |
| PEEP | 5 | 5 | 8 | 8 | 10 | 10 | 10 | 12 | 14 | 14 | 14 | 16 | 18 | 18-24 |
| High PEEP protocol used in the ALVEOLI study | | | | | | | | | | | | | | |
| FiO_2_ | 0.3 | 0.3 | 0.4 | 0.4 | 0.5 | 0.5 | 0.5-0.8 | | 0.8 | 0.9 | 1.0 | | | |
| PEEP | 12 | 14 | 14 | 16 | 16 | 18 | 20 | | 22 | 22 | 22-24 | | | |

The ALVEOLI study randomized patients into low and high PEEP protocols. There was a protocol change in the high PEEP arm of the study, and the current table shows the subsequent protocol. The ALTA study allowed a low PEEP or a high PEEP protocol or clinician discretion.

Definition of abbreviations: FiO_2_ = fraction of inspired oxygen; PEEP = positive end-expiratory pressure in cmH_2_O.

**Table E3. Pairwise comparisons of areas under the receiver operating characteristic curves for different PEEP thresholds**

| Comparisons of AUC for different PEEP thresholds | P value for comparisons of AUC using the P/F ratio | P value for comparisons of AUC using the P/FP ratio |
| --- | --- | --- |
| ≥ 5 versus > 5 cmH_2_O | < 0.001 | < 0.001 |
| > 5 versus > 8 cmH_2_O | 0.057 | < 0.001 |
| > 8 versus > 10 cmH_2_O | 0.149 | < 0.001 |
| > 10 versus > 12 cmH_2_O | 0.719 | 0.119 |
| > 12 versus > 14 cmH_2_O | 0.060 | < 0.001 |
| > 14 versus > 16 cmH_2_O | 0.112 | 0.013 |
| > 16 versus > 18 cmH_2_O | 0.873 | 0.374 |

Definition of abbreviations: AUC = area under the curve; PEEP = positive end-expiratory pressure; P/F = ratio of the partial pressure of arterial oxygen (PaO_2_) to the fraction of inspired oxygen (FiO_2_); P/FP = (PaO_2_ * 10)/(FiO_2_ * PEEP).

**Figure E1. Included patients from seven National Heart, Lung, and Blood Institute (NHLBI) ARDS Clinical Trials Network studies**


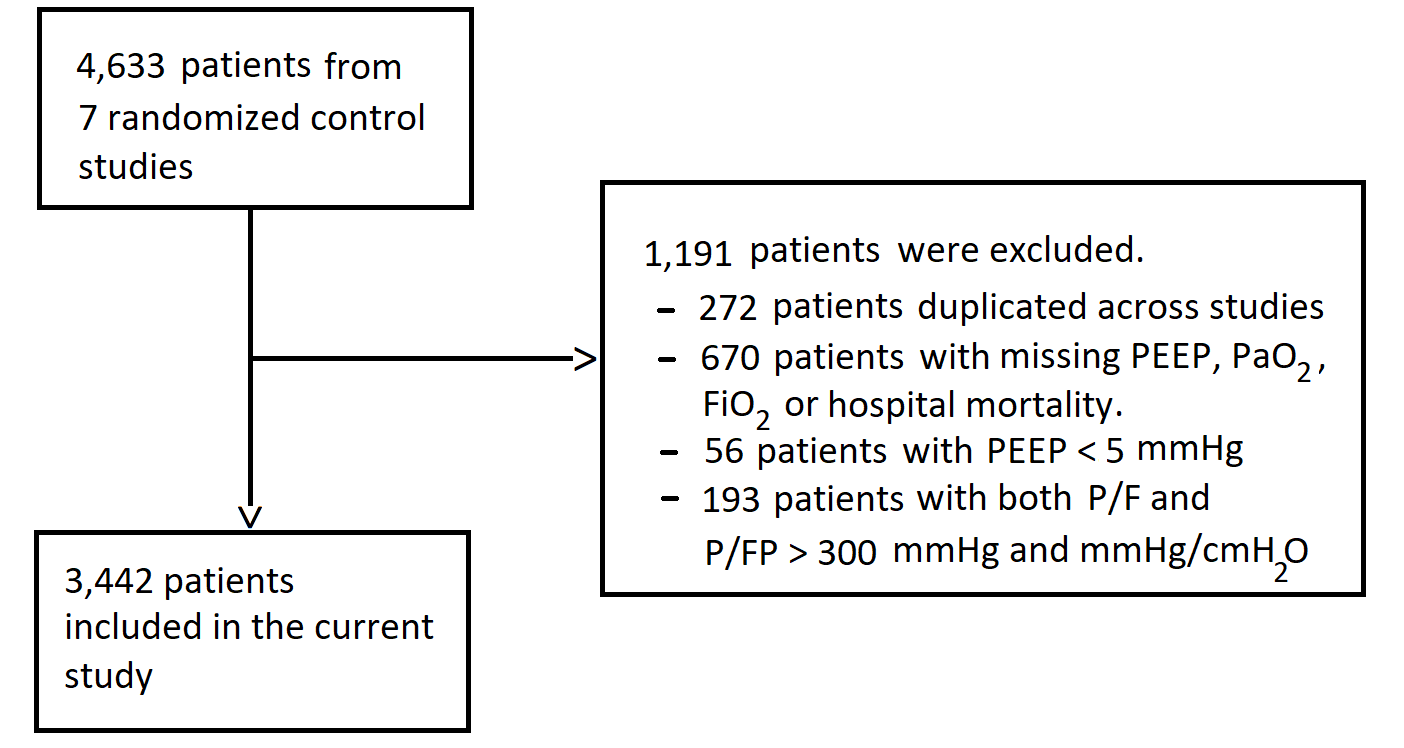


Definition of abbreviations: ARDS = acute respiratory distress syndrome; FiO_2_ = fraction of inspired oxygen; PaO_2_ = partial pressure of arterial oxygen; PEEP = positive end-expiratory pressure; P/F = ratio of the PaO_2_ to FiO_2_; P/FP = (PaO_2_ * 10)/(FiO_2_ * PEEP).

**Figure E2.** **Regression line of PEEP versus P/F ratio**


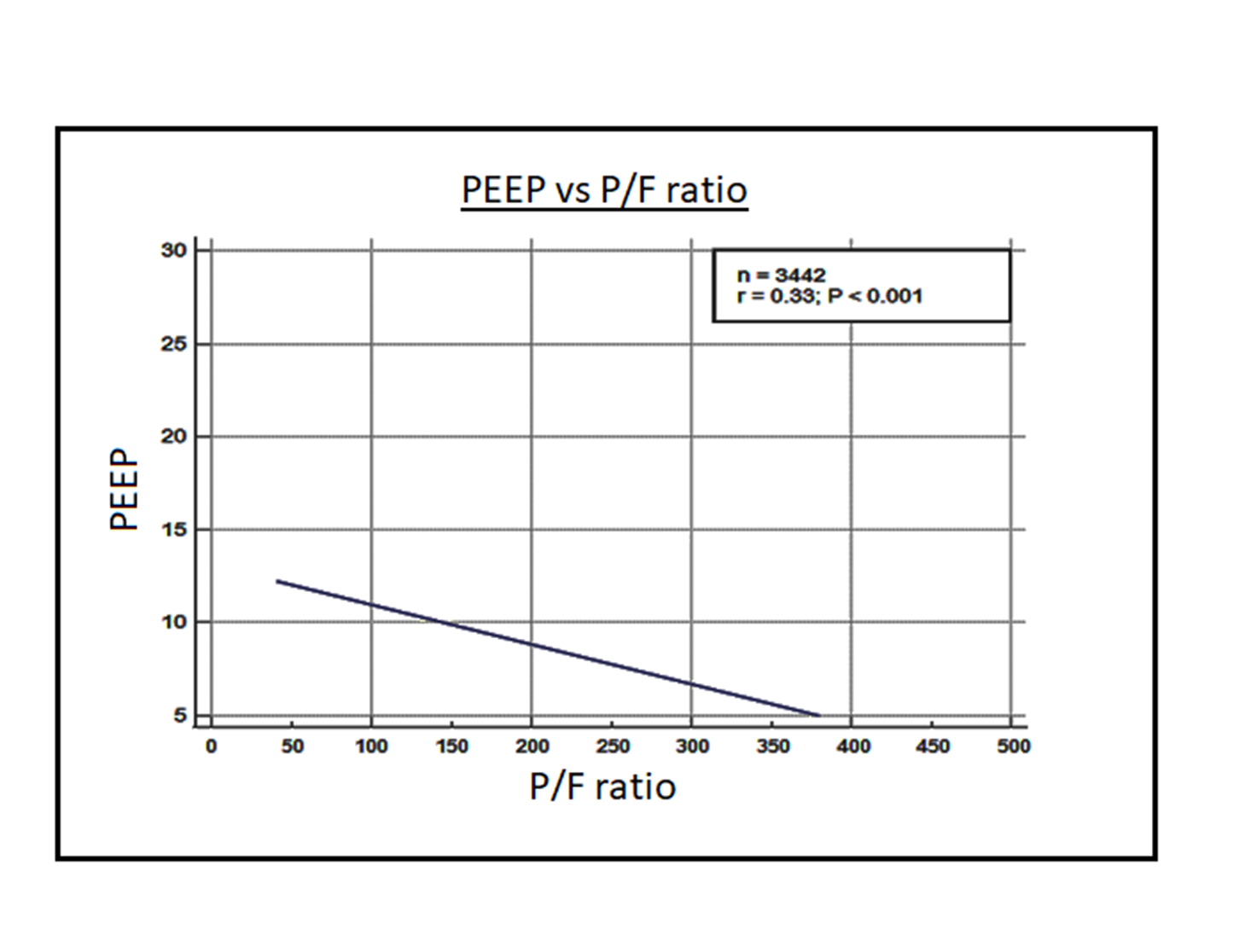


The regression line intersects the P/F ratio of 150 mmHg at a PEEP of 10 cmH_2_O.

Definition of abbreviations: P/F = ratio of the partial pressure of arterial oxygen (PaO_2_) to the fraction of inspired oxygen (FiO_2_); P/FP = (PaO_2_ * 10)/(FiO_2_ * positive end-expiratory pressure).

**Figure E3. Change of severity classifications when P/FP ratio is used instead of P/F ratio**


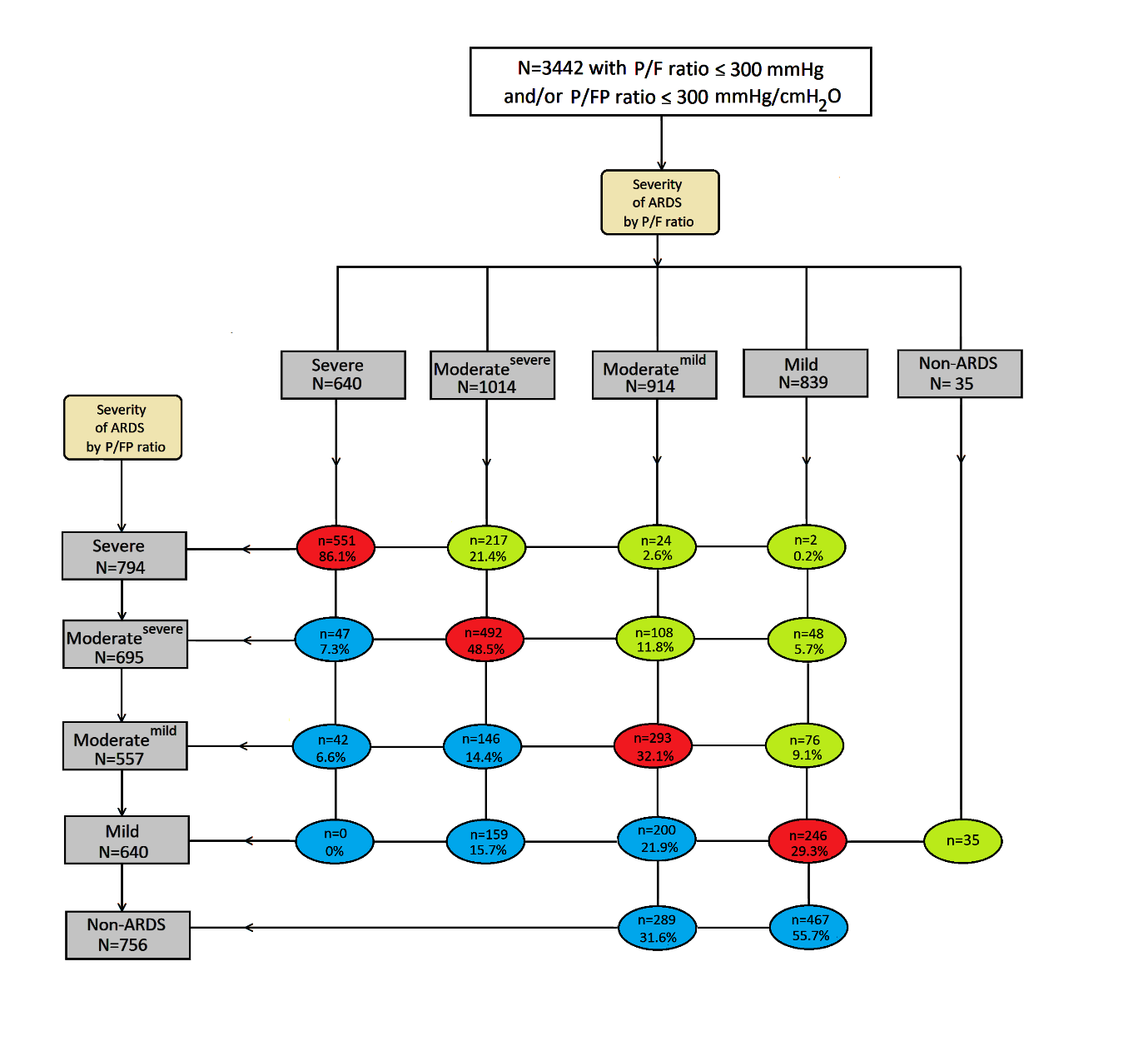


Severe refers to a ratio of ≤ 100, moderate ^Severe^ refers to a ratio of 101-150, moderate ^mild^ refers to a ratio of 151 to 200 and mild refers to a ratio of 201-300, non-ARDS refers to a ratio of > 300 mmHg or mmHg/cmH_2_O. Green ovals represent patients who were reclassified to a more severe category. Blue ovals represent patients who were reclassified to a milder category. Red ovals represent patients whose categories remained unchanged.

Definition of abbreviations: ARDS = acute respiratory distress syndrome; P/F = ratio of the partial pressure of arterial oxygen (PaO_2_) to the fraction of inspired oxygen (FiO_2_); P/FP = (PaO_2_ * 10)/(FiO_2_ * positive end-expiratory pressure).

**Figure E4. Scatter plots of patients according to P/F ratio and P/FP ratio**


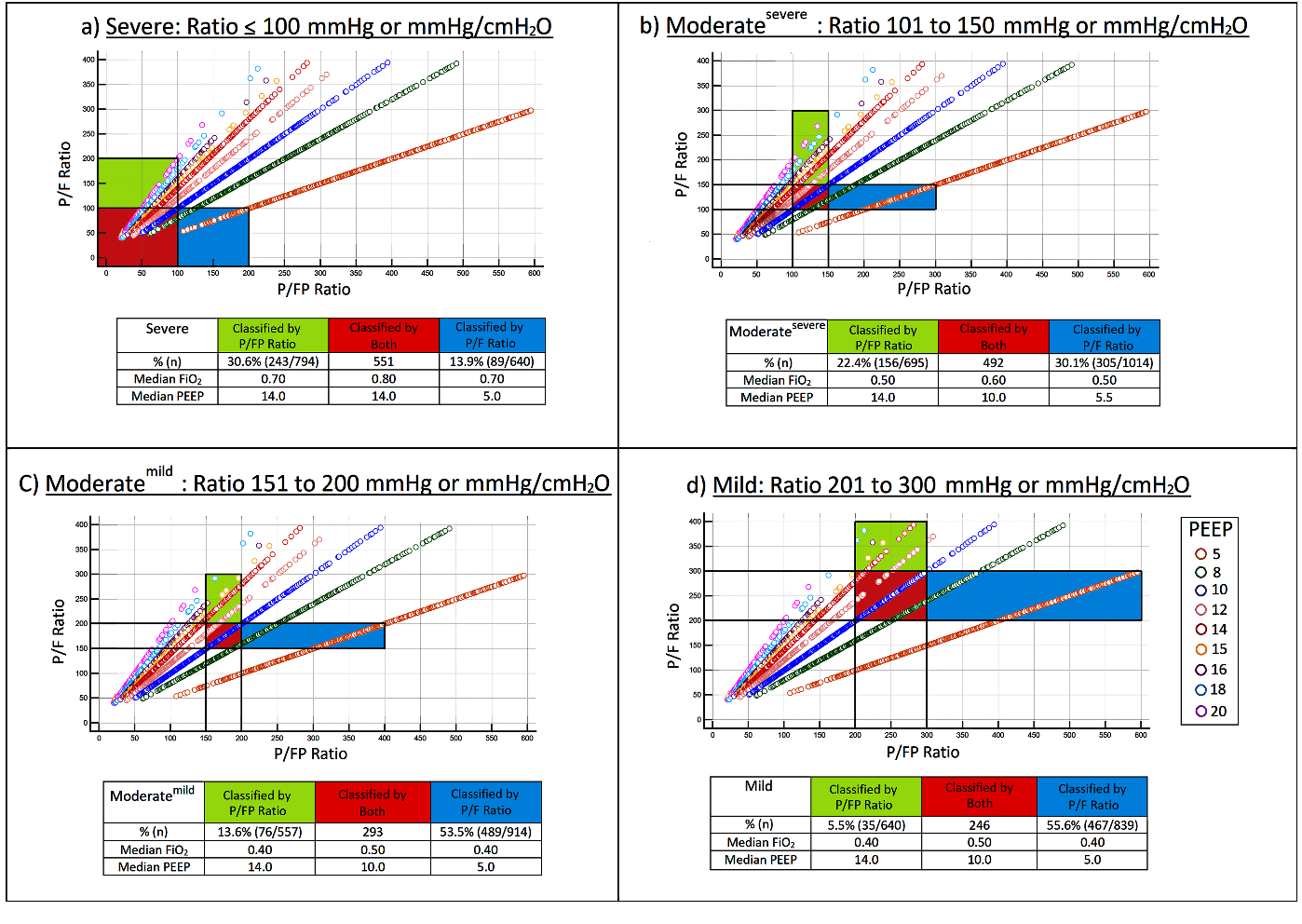


Each circle represents individual patients on a certain applied PEEP; each colour represents a different PEEP setting. Green bars represent patients who were classified by the P/FP ratio in each severity. Blue bars represent patients who were classified by the P/F ratio in each severity. Red bars represent patients whose categories remained unchanged.

Definition of abbreviations: FiO_2_ = fraction of inspired oxygen; PEEP = positive end-expiratory pressure; P/F = ratio of the partial pressure of arterial oxygen (PaO_2_) to FiO_2_; P/FP = (PaO_2_ * 10)/(FiO_2_ * PEEP).

**Figure E5. Change of severity classifications when P/FP ratio is used instead of P/F ratio in the low PEEP arm of the ALVEOLI study**


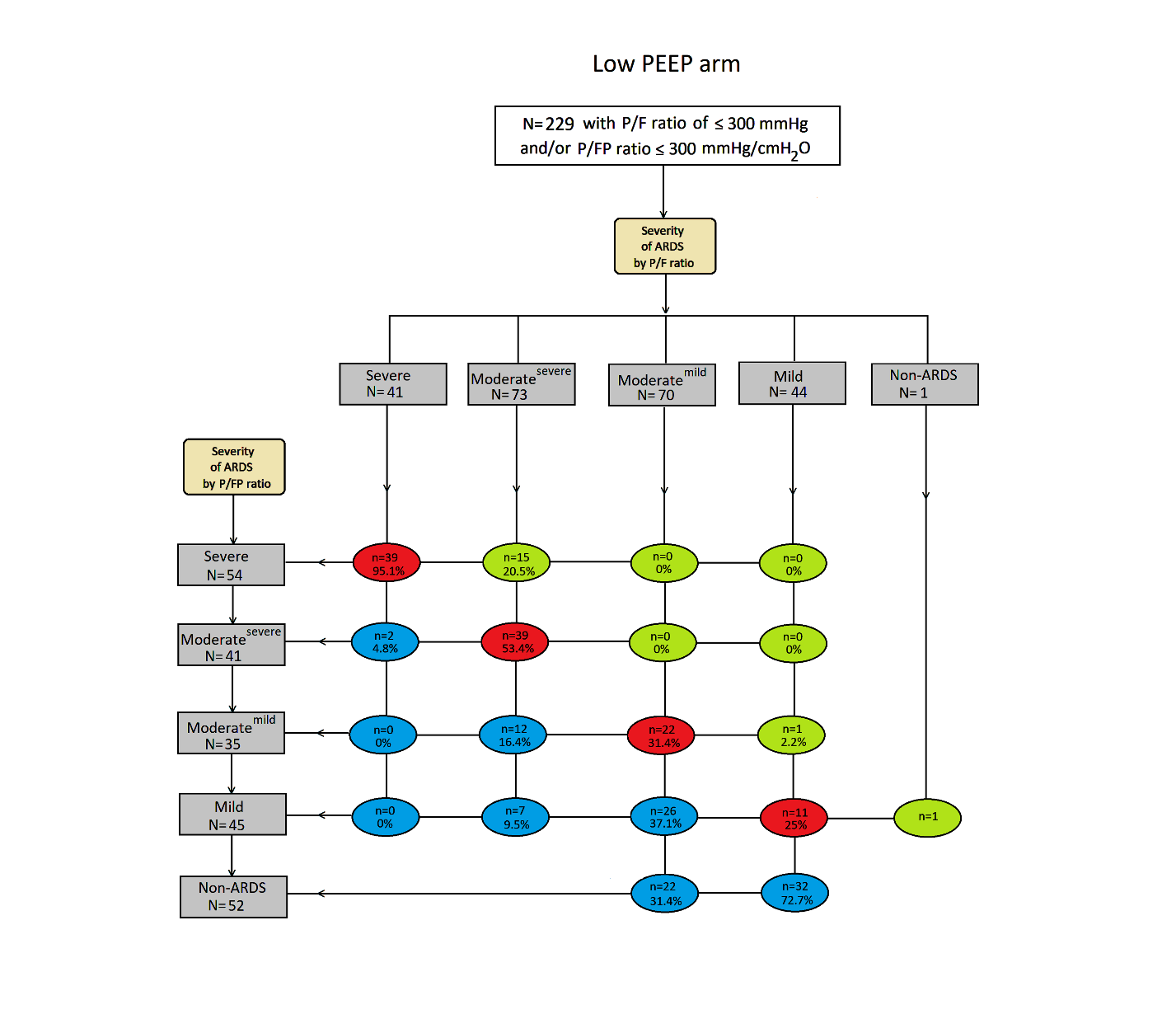


Flowchart for the movement of patients from the P/F ratio to the P/FP ratio. Green ovals represent patients who were reclassified to a more severe category. Blue ovals represent patients who were reclassified to a milder category. Red ovals represent patients whose categories remained unchanged. Non-ARDS refers to patients with P/F or P/FP ratio > 300 mmHg or mmHg/cmH_2_O.

Definition of abbreviations: ARDS = acute respiratory distress syndrome; P/F = ratio of the partial pressure of arterial oxygen (PaO_2_) to the fraction of inspired oxygen (FiO_2_); P/FP = (PaO_2_ * 10)/(FiO_2_ * positive end-expiratory pressure).

**Figure E6. Change of severity classifications when P/FP ratio is used instead of P/F ratio in the high PEEP arm of the ALVEOLI study**


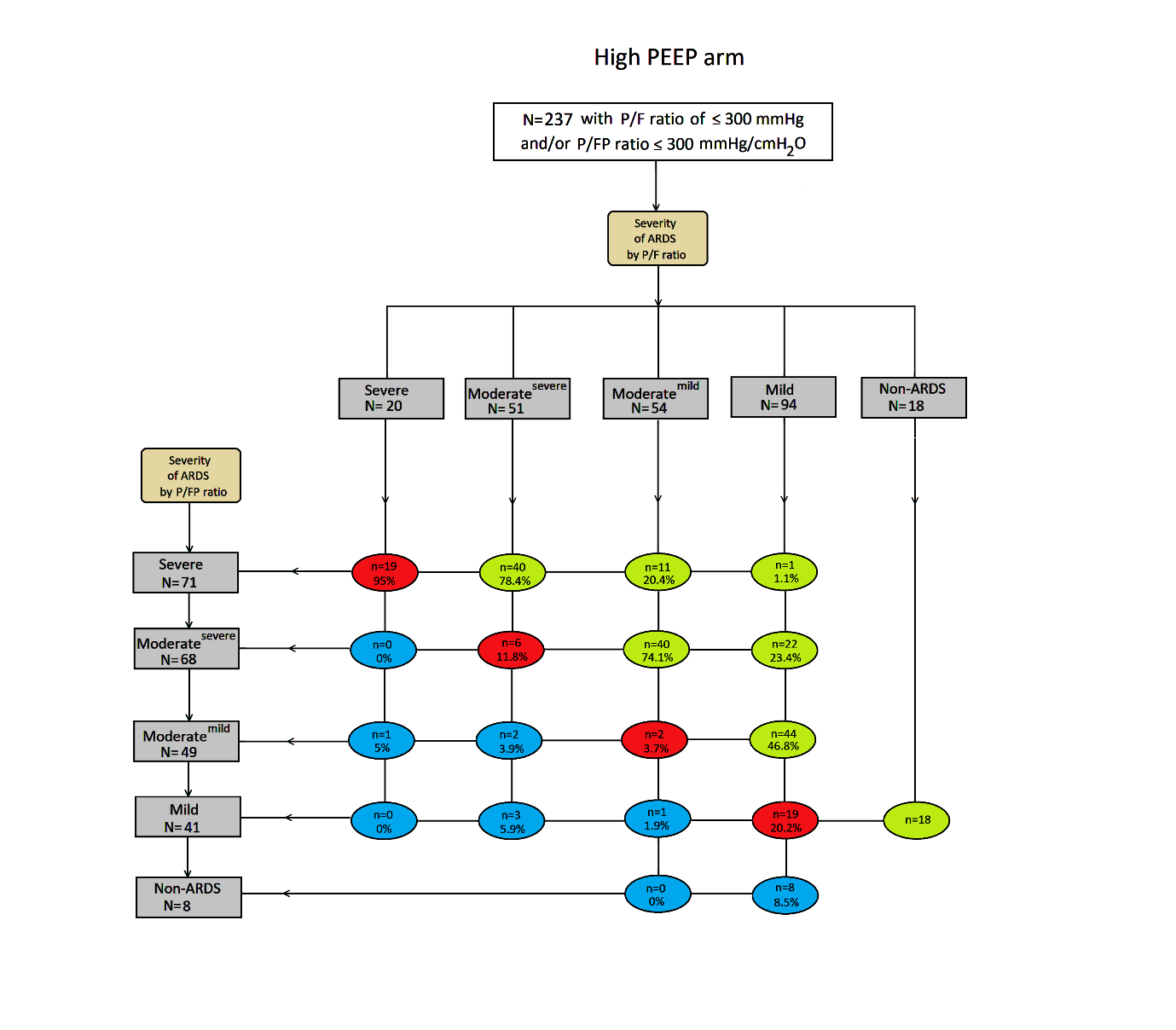


Flowchart for the movement of patients from the P/F ratio to the P/FP ratio. Green ovals represent patients who were reclassified to a more severe category. Blue ovals represent patients who were reclassified to a milder category. Red ovals represent patients whose categories remained unchanged. Non-ARDS refers to patients with P/F or P/FP ratio > 300 mmHg or mmHg/cmH_2_O.

Definition of abbreviations: ARDS = acute respiratory distress syndrome; P/F = ratio of the partial pressure of arterial oxygen (PaO_2_) to the fraction of inspired oxygen (FiO_2_); P/FP = (PaO_2_ * 10)/(FiO_2_ * positive end-expiratory pressure).

**Figure E7. Comparison of P/FP ratios using different correction factors**


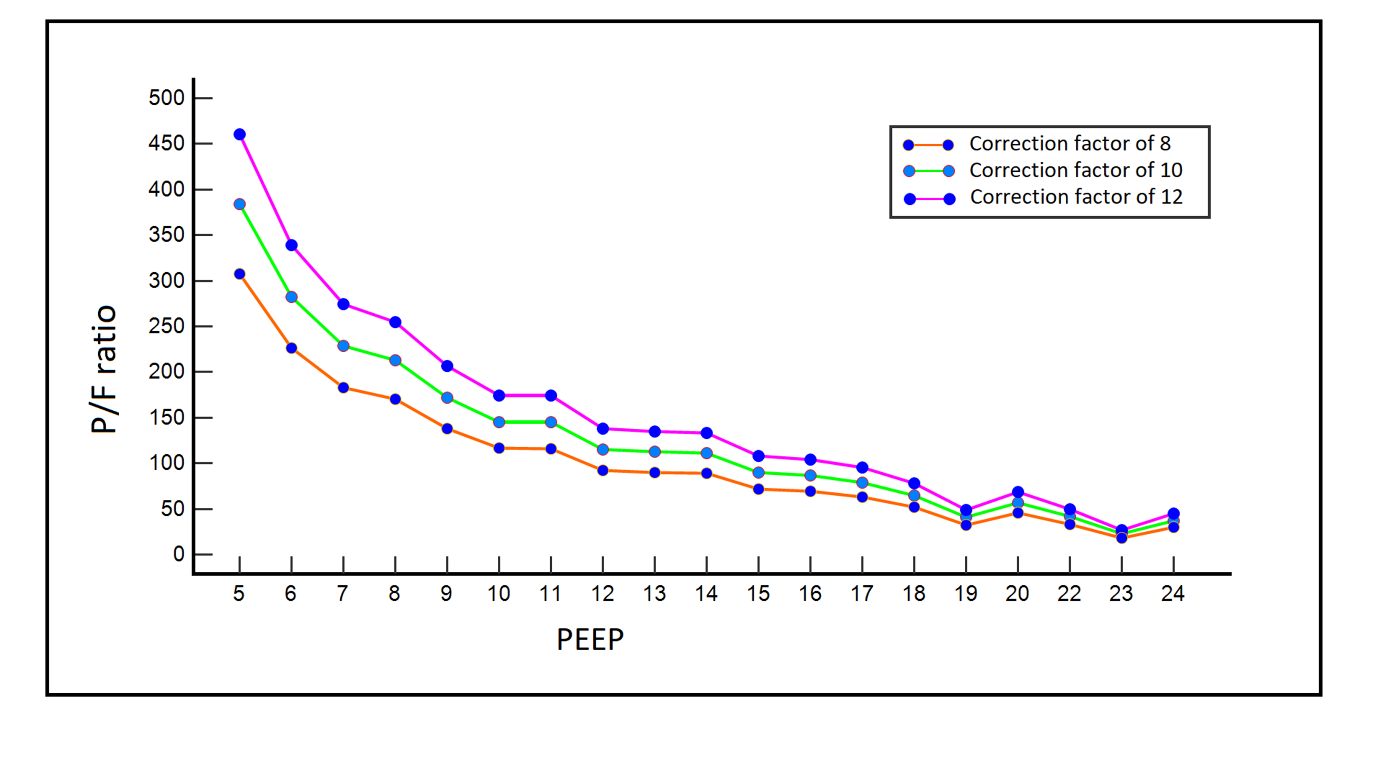


Each curve represents a different correction factor: 8, 10, and 12. A correction factor of 10 is used throughout the manuscript, i.e. P/FP = (PaO_2_ * 10)/(FiO_2_ * positive end-expiratory pressure). To derive the other curves, correction factors of 8 and 12 are used instead.

Definition of abbreviations: P/F = ratio of the partial pressure of arterial oxygen (PaO_2_) to the fraction of inspired oxygen (FiO_2_).
